# Autoimmune Implications in a Patient with Graves’ Hyperthyroidism, Pre-eclampsia with Severe Features, and Primary Aldosteronism

**DOI:** 10.3390/medicina60010170

**Published:** 2024-01-17

**Authors:** Benjamin Lin, Lauren Robinson, Basem Soliman, Jill Gulizia, Stephen Usala

**Affiliations:** 1 School of Medicine, Texas Tech University Health Sciences Center, Lubbock, TX 79430, USA; ben.lin@ttuhsc.edu (B.L.); lauren.h.robinson@ttuhsc.edu (L.R.); 2 Department of Surgery, Texas Tech University Health Sciences Center, Amarillo, TX 79106, USA; basem.soliman@ttuhsc.edu; 3 Women’s Healthcare Associates, Obstetrics and Gynecology, Amarillo, TX 79106, USA; jgulizia@whaonline.net; 4 Department of Internal Medicine, Texas Tech University Health Sciences Center, Amarillo, TX 79106, USA

**Keywords:** Graves’ disease, aldosteronism, autoimmune, pre-eclampsia

## Abstract

*Background and Objectives*: Graves’ disease (GD) and primary aldosteronism (PA) are two pathologies that can cause significant morbidity and mortality. GD is mediated by autoantibodies, and recent studies have shown autoantibody involvement in the pathophysiology behind both PA and pre-eclampsia. The coexistence of GD and PA, however, is reportedly rare. This report describes a unique case of Graves’ hyperthyroidism and concomitant PA in a patient with a history of pre-eclampsia with severe features. *Case Presentation*: The patient presented at 17 weeks pregnancy with mild hyperthyroidism, negative TSH receptor antibodies, and a low level of thyroid-stimulating immunoglobulins (TSI). Her TSH became detectable with normal thyroid hormone levels, and therefore, no anti-thyroid medication was administered. At 34 weeks she developed pre-eclampsia with severe features, and a healthy child was delivered; her TSH returned to normal. Seven months after delivery, she presented emergently with severe hyperthyroidism, hypertensive crisis, and a serum potassium of 2.5 mmol/L. Her hypertension was uncontrolled on multiple anti-hypertensives. Both TSI and TSH receptor antibodies were negative. The aldosterone(ng/dL)/renin(ng/mL/h ratio was (13/0.06) = 216.7, and abdominal CT imaging demonstrated normal adrenal glands; thus, a diagnosis of PA was made. Her blood pressure was subsequently controlled with only spironolactone at 50 mg 2xday. Methimazole was started but discontinued because of an allergic reaction. Consequently, a thyroidectomy was performed, and pathology revealed Graves’ disease. The patient remained well on levothyroxine at 125 mcg/day and spironolactone at 50 mg 2xday three months after the thyroidectomy. *Conclusions*: This patient manifested severe GD with antibodies undetectable by conventional TSI and TSH receptor assays and accelerated hypertension from PA simultaneously. These conditions were successfully treated separately by spironolactone and thyroidectomy. Autoimmune PA was considered likely given the clinical picture. The diagnosis of PA should be considered in hypertension with GD.

## 1. Introduction

Graves’ disease (GD) is an autoimmune disease of the thyroid gland. It is the most common cause of hyperthyroidism in developed countries [1], affecting 2% of women and 0.5% of men globally [2], and is associated with adverse effects on the quality of life [3]. In GD, autoantibodies (TRAb) against the thyroid-stimulating hormone receptor (TSHR) induce excess thyroid hormone secretion, which can cause severe hyperthyroidism with associated tachycardia, hypertension, fever, delirium, and an increased risk of death [2]. Recent studies have shown that other common diseases, such as primary aldosteronism (PA) and pre-eclampsia, may also have autoimmune-related mechanisms that underlie their pathogenesis [4,5]. PA is the most common form of secondary hypertension (HTN), accounting for 5–10% of all patients and increasing in prevalence in patients with severe or treatment-resistant hypertension [6]. Compared to patients with primary HTN, patients with PA have an increased risk of cardiovascular complications [7]. Pre-eclampsia is an enigmatic, hypertensive disease and a leading cause of fetal and maternal morbidity and mortality [8,9]. Stimulating autoantibodies (AT1R-Abs) against the angiotensin II type 1 receptor (AT1R) have been implicated in both PA and pre-eclampsia and may play a role in their pathogenesis [5]. Concomitant GD and PA as well as PA in pregnancy are both reportedly rare combinations [10,11]. This study therefore presents a unique case of a patient with GD, PA, and a prior history of pre-eclampsia with severe features, as well as an overview of the literature describing the autoimmune nature of these three diseases. 

## 2. Detailed Case Description

The patient was referred to the endocrine clinic (30 June, Table 1) for hyperthyroidism and pregnancy of 17 weeks. She had been diagnosed with hypothyroidism and treated with levothyroxine for two years but had not been on thyroid hormone for the past five years. One month prior to the initial endocrine visit, symptoms of feeling hot and tremulous as well as a racing heart had resulted in an emergency room visit where hyperthyroidism was diagnosed. In clinic, her pulse rate was 98, BP was 124/62, weight was 93 kg (205 lbs), and height was 157 cm (5 feet, 2 inches). No goiter was observed. Thyroid function tests revealed the following: TSH, 0.00 μIU/mL (0.00 mIU/L); Free Thyroxine (Free T4), 0.7 ng/dL (9.0 pmol/L); and Free Triiodothyronine (Free T3), 4.61 pg/mL (7.08 pmol/L). The TSH receptor antibody assay was negative. The TSI (thyroid-stimulating immunoglobulins) assay was weakly positive at 163% (normal <140%), and a thyroid sonogram showed no nodules but did show a heterogeneous echotexture. A diagnosis of mild Graves’ hyperthyroidism was made. The patient was seen in the endocrine clinic every two to four weeks, and no anti-thyroid medication was initiated because of the following: the TSH became detectable, the Free T3 was only minimally elevated, the TSI was normal to only mildly elevated, and there was no fetal distress.

During her pregnancy, the patient developed gestational diabetes, requiring insulin and polyhydramnios. The patient’s blood pressure was normal at multiple endocrine clinic visits (BP (120–124)/(62–68)) until October (27 October, Table 1 and Table 2), when she developed pre-eclampsia with severe features. During her hospitalization, her blood pressures were (164–177)/(84–102). She required IV Labetalol for severe-range blood pressures during her labor course. A healthy 4.080 kg male, Apgar 8,9, was delivered at 34 and 4/7 weeks, and she did not require anti-hypertensives at discharge. The gestational diabetes resolved, but subsequent mild hypertension (BP ~144/88) was treated by her obstetrician and primary care physician using lisinopril and hydrochlorothiazide. When seen in clinic 2.5 months (12 January) after delivery, her BP was 140/90 on lisinopril and hydrochlorothiazide, and her TSH and Free T4 were normal, at 2.1 μIU/mL (or mIU/L) and 0.9 ng/dL (11.58 pmol/L), respectively.

However, the patient’s hypertension increased 6.5 months after pre-eclampsia and delivery, and the patient went to the emergency room for paresthesia and weakness of her right upper extremity (15 May, Table 1). She was found to be hypertensive, with a BP of 169/98 and pulse rate of 97, and hypokalemic, with a serum potassium of 2.5 meq/L (mmol/L). A brain CT revealed no cranial hemorrhage. She was treated with intravenous potassium and hydralazine, and oral potassium chloride was added to her lisinopril and hydrochlorothiazide. In addition, she was found to be quite hyperthyroid, with a TSH of 0.00 (μIU/mL or mIU/L), Free T4 of 3.93 ng/dL (50.57 pmol/L), and Free T3 of 14.62 pg/mL (22.45 pmol/L), and was referred for a follow-up with the endocrine clinic. The diagnosis of PA was entertained and evaluated by the aldosterone-to-renin ratio (ARR). The ARR at a potassium of 2.7 meq/L (mmol/L) was (13 ng/dL)/(0.06 ng/mL/h) = 216.7, and at a potassium of 3.2 meq/L (mmol/L), was (9 ng/dL)/(0.07 ng/mL/h) = 128.6. Spironolactone 50 mg 2x/day was started, and she was weaned off the potassium chloride, hydrochlorothiazide, lisinopril, and hydralazine. The systolic BP seven days after starting the spironolactone was 118 mm, and two weeks later the BP was 124/76. Her hypokalemia had resolved with a potassium of 3.6 meq/L(mmol/L) off of any potassium chloride supplementation.

The patient’s hyperthyroidism was treated with Methimazole, but it was discontinued because of a serious rash (Table 1 and Table 2). Two TSI measurements were in the normal range and a TSH receptor antibody level was negative. The patient was given a brief course of dexamethasone, and a thyroidectomy was necessary to treat the severe hyperthyroidism. The surgical pathology confirmed the diagnosis was Graves’ disease in the context of negative TSI and TSH receptor antibody assays. The patient reported immediate relief after thyroidectomy, but unadvisedly stopped her spironolactone for 10 days. This resulted in recurrent hypertension (BP 160 s/100–110) and hypokalemia (potassium of 3.2 meq/L (mmol/L)). Spironolactone at 50 mg 2xday was restarted with the resolution of the hypertension and hypokalemia. Post-surgical hypothyroidism was treated with levothyroxine.

The patient was seen again three months after thyroidectomy and continued well on levothyroxine at 125 mcg/day with a normal BP and serum potassium on spironolactone at 50 mg 2xday.

## 3. Discussion

Recent studies have shown associations between primary aldosteronism (PA) and several thyroid pathologies. PA has been associated with increased thyroid abnormalities or alterations in ultrasonography including nodules, multinodular goiter, and papillary carcinoma [14,15]. PA has also been linked to increased thyroid dysfunction and increased anti-thyroid autoantibodies (anti-thyroid peroxidase and anti-thyroglobulin) [16,17]. Several case reports have described PA complicated by autoimmune Hashimoto’s thyroiditis [18,19], with one case noting improvement in hypothyroid symptoms after the medical and surgical treatment of PA [19]. The association between Graves’ disease (GD) and PA has not been well studied. Several case reports have documented concomitant PA and Graves’ hyperthyroidism resulting in either thyrotoxicosis or hypokalemic paralysis [10,20,21]. Other reports have shown coexisting PA with other forms of hyperthyroidism, including toxic nodular goiter or unspecified hyperthyroidism, with consequent hypokalemic paralysis [10,22]. While there is a paucity of literature describing the association between GD and PA, there is increasing evidence on the role of PA in thyroid disease. Some studies have proposed autoimmune processes as an underlying cause, as stimulatory autoantibodies exist in both GD and PA. However, the exact mechanism remains unclear and requires further investigation [10,19]. 

The autoimmune nature of both PA and pre-eclampsia revolves around autoantibodies against the angiotensin II type 1 receptor (AT1R) [5,23]. The AT1R is a G-protein-coupled receptor (GPCR) widely distributed throughout the human body and plays a significant role as part of the renin–angiotensin–aldosterone system (RAAS) in blood pressure regulation, vasoconstriction, inflammation, and cardiovascular functioning [23]. Autoantibodies against the AT1R (AT1R-Abs) have been implicated in various pathologies, including pre-eclampsia, PA, renal graft failure, and systemic sclerosis [5,24]. AT1R-Abs activity has been shown in patients with PA due to aldosterone-producing adenomas (APAs) and bilateral adrenal hyperplasia (BAH) [25], with significantly increased activity when compared to normotensive controls [26]. Kem et al. showed that serum from patients with PA significantly increased AT1R activation in AT1R-transfected cells compared to serum from normotensive controls, with activity inhibited by the AT1R blocker losartan [27]. The same study showed that IgG from AT1R-Abs-positive sera yielded vasoconstrictive effects in rat cremaster arterioles, with activity blocked by losartan, and stimulated aldosterone production in cultured adrenal cells, with activity blocked by candesartan. Aldosterone production was enhanced with the addition of angiotensin II, suggesting an AT1R-Abs-mediated allosteric conformational change in the AT1R that facilitates angiotensin II binding [27]. 

The mechanism of the relationship between autoimmune-induced PA and the conventional view of PA as APA and BAH has yet to be elucidated. Aldosterone-producing cell clusters, now termed multiple aldosterone-producing nodules or micronodules, are felt to be the precursors of APA and BAH [28,29,30]. Interestingly, one study of 107 adrenal glands of normotensive Japanese patients from an 837-autopsy consecutive cohort revealed that aldosterone-producing cell clusters are common, with 34% harboring known aldosterone-driver mutations [31]. One hypothesis proposes that AT1R-Abs play a role in the chronic stimulation of the zona granulosa, with a consequent hyperproliferative state potentially enabling the development of APA or BAH [5]. These studies show increasing evidence for an autoimmune mechanism involving AT1R-Abs that may underlie PA development.

AT1R-Abs have also been implicated in pre-eclampsia. Multiple studies have shown a high prevalence of AT1R-Abs in pre-eclampsia [5,32]. AT1R-Abs can be detected in the second trimester and may be an early but nonspecific marker for pre-eclampsia [33]. AT1R-Abs titers have a strong positive correlation with the severity of pre-eclampsia [34], and antibody activity decreases after delivery but does not regress completely [32,35]. Zhou et al. provided strong evidence for pre-eclampsia as a pregnancy-induced autoimmune disease by injecting AT1R-Abs from pre-eclamptic women into pregnant mice, which induced pre-eclamptic features such as hypertension, proteinuria, glomerular endotheliosis, placental abnormalities, and decreased fetal size [36]. These features were blocked with losartan and a synthetic blocking peptide. Further studies reinforced these findings by inducing pre-eclamptic symptoms in pregnant mice via the injection of AT1R-Abs [37,38,39]. Several studies showed upregulated endothelin-1 activity and decreased mean arterial pressure (MAP) in response to endothelin-1 antagonists [37,38]. One study by Wenzel et al. showed significant increases in MAP when AT1R-Abs were combined with angiotensin II, suggesting an AT1R-Abs-mediated increase in the sensitivity of the AT1R [39]. This is in line with the results of Kem et al. in their investigation of the role of AT1R-Abs in PA [27]. The aforementioned studies underscore the potential role of AT1R-Abs and autoimmune processes in the pathogenesis of both pre-eclampsia and PA.

In this patient with acute, severe illness from both GD and PA, and a prior history of pre-eclampsia with severe features, it is important to highlight the common presence of stimulatory autoantibodies in GD, PA, and pre-eclampsia. Autoantibodies to the thyroid-stimulating hormone receptor (TRAbs to the TSHR) are present in the vast majority of GD cases. Autoantibodies to the AT1R are present in both PA and pre-eclampsia and may play a role in inducing pathological symptoms for both diseases. However, in this patient, there are predominantly negative antibody titers for TRAb, as measured by both thyroid-stimulating immunoglobulin (TSI) and thyrotropin-binding inhibitory immunoglobulin (TSH receptor antibodies/TBII) assays. Modern third-generation TRAb assays have a sensitivity and specificity in the upper 90% for patients with overt, clinical GD hyperthyroidism [40]. In patients with subclinical or mild GD hyperthyroidism, the TRAb test is less reliable and is more often negative [41]. In such cases with negative TRAb titers, Doppler ultrasonography or thyroid scintigraphy can detect nodules, vascularity, and radioactive uptake that can help differentiate GD from other forms of hyperthyroidism [42]. Post-thyroidectomy histology for GD with negative TRAbs often show marked or moderate lymphocytic infiltration [43]. However, in contrast, this patient demonstrated a severe GD phase with negative TRAb tests, and furthermore, surgical pathology showed only patchy mild chronic thyroiditis but with diffuse follicular hyperplastic changes. These features indicate that this case is an atypical presentation of GD in the context of PA. Given the coexistence of GD, PA, and prior pre-eclampsia with severe features in this patient, as well as known autoimmune mechanisms for GD, PA, and pre-eclampsia, it is tempting to suggest an unexplored related autoimmune connection or pathway. While AT1R-Abs may be involved in the pathogenesis of PA and pre-eclampsia, and AT1R is present in thyroid tissue, a mechanism to explain a connection has not been elucidated.

As exemplified in this presented case, the clinical treatment of coexisting GD and PA can be performed successfully by treating each disease separately. Standard anti-thyroid medication may be administered, with thyroidectomy as an alternative if contraindications for medical therapy arise [2,3]. Depending on the presence of an aldosterone-producing adenoma or bilateral (idiopathic) adrenal hyperplasia (the two most common etiologies of PA) [44], adrenalectomy or mineralocorticoid antagonists (spironolactone or eplerenone) may be considered, respectively [12,13]. Ultimately, medical or surgical therapy for GD and PA should be tailored to each individual patient and based on robust empirical evidence. Finally, PA should be considered in the onset of new or uncontrolled hypertension in GD.

The presented case report has some limitations. It is a retrospective analysis of a single patient and therefore difficult to generalize to a larger population. The use of AT1R and AT1R-Ab assays to explore the molecular pathology was beyond the scope of this case report. Investigating the role of AT1R-Abs in patients with GD and hypertension may assist in understanding potential connections between GD, PA, pre-eclampsia, and other autoimmune endocrinopathies. 

## 4. Conclusions

In this female patient, following pre-eclampsia with severe features, there was the simultaneous development of severe GD and accelerated hypertension from PA. An unusual antibody mechanism was indicated by the absence of the typical TSI and TSH receptor antibodies for GD. Imaging revealed no adrenal adenoma, and autoimmune-driven PA was considered likely. This case shows the successful long-term treatment of coexistent severe GD and PA by thyroidectomy and spironolactone. PA should be considered in new or uncontrolled hypertension in GD. 

## Figures and Tables

**Table 1:** Timeline of Graves’ hyperthyroidism during pregnancy, delivery, and thyroidectomy.

Date	Symptoms and Signs Clinical Status	TSH (0.45–5.33 μIU/mL) ^1^	Free T4 (0.58–1.64 ng/dL) ^1^	Free T3 (2.50–3.90 pg/mL) ^1^	TSI ^2^ (<140%)	Thyroid Treatment
30 Jun	Tremulousness, overheated, racing heart; 17 weeks pregnant	0	0.89	4.7	163%; TSH Receptor Antibodies negative	none
8 Jul		0	0.7	4.61		
30 Aug		0.03	0.5	4.03		
12 Sep	Thyroid sonogram: no nodules					
1 Oct	Oral glucose tolerance test positive for gestational diabetes; treated with diet and later insulin	0.04	0.47	3.57	102%	
27 Oct	^3^ Preeclampsia with severe features; Cesarean delivery 34 4/7 weeks; male, 4.080 kg, Apgar 8, 9; gestational diabetes resolved 6 weeks postpartum					
12 Jan		2.1	0.9			
15 May	Emergency room for paresthesia and weakness of right arm, BP 169/98, pulse 97	0	3.93	14.62	121%	Started: Methimazole: 10mg 3x/day, Metoprolol: 25mg 2x/day, Dexamethasone: 1mg, 2x/day × 7days
31 May		0	1.75	4.94		
6 Jun	Rash	0	1.26	5.61		Stopped: Methimazole
20 Jun		0	2.24	10.03	124%	
25 Jul		0	3.42	13.8	TSH Receptor Antibodies negative	Dexamethasone 1mg, 2x/day × 7days
12 Aug	Diffuse follicular hyperplastic changes with patchy mild chronic thyroiditis on pathology; pathological diagnosis, Graves’ Disease					Thyroidectomy, Started: Levothyroxine 125mcg/day
31 Aug		0.01	0.87	3.98		Levothyroxine 125mcg/day

^1^ TSH (Thyroid Stimulating Hormone): 1 μIU/mL = 1 mIU/L; Free T4 (Free Thyroxine): 1 ng/dL = 12.87 pmol/L; Free T3 (Free Triiodothyronine): 1 pg/mL = 1.536 pmol/L. ^2^ TSI (Thyroid Stimulating Immunoglobulins); TSH Receptor Antibodies were also measured; ^3^ During her preeclampsia with severe features, BP ranged (164–177)/(84–102), requiring IV Labetalol. BP treatment was not required immediately postpartum.

**Table 2:** Timeline of primary aldosteronism (PA) during pregnancy, delivery, and thyroidectomy.

Date	BP and Potassium (meq/L) ^1^	Medications and Clinical Status	Aldosterone/Renin Ratio (ARR) (ng/dL)/(ng/mL/h) ^2^
30 Jun	124/62	none	
27 Oct	(164–177)/(84–102)	^3^ Preeclampsia with severe features; Delivery	
12 Jan	140/90	Lisinopril Hydrochlorothiazide	
15 May	169/98 Potassium 2.5	Lisinopril Hydrochlorothiazide Added: Hydralazine and Potassium Chloride	
25 May 31 May	Potassium 2.7 Potassium 3.2		13/0.06 = 216.7 9/0.07 = 128.6
6 Jun	162/90	Started: Spironolactone: 50mg 1x/day, three days later increased 2xday. Stopped: Potassium Chloride and BP medications	
13 Jun	118 systolic by palpation		
20 Jun	124/76 Potassium 3.6		
25 Jul	130/74		
12 Aug	130/80	Thyroidectomy Started: Levothyroxine	
22 Aug	160s/100–110 Potassium 3.2	Patient had stopped spironolactone. It was restarted, 50mg 2x/day	Patient had discontinued spironolactone for 10 days. Repeat A/R: 6/0.25 = 24.0
31 Aug	130/74 Potassium 4.2	Spironolactone, 50mg, 2x/day	

^1^ Serum potassium: 1 meq/L = 1 mmol/L; ^2^ Aldosterone: conventional units 1 ng/dL = 27.7 pmol/L; Renin: conventional units 1 ng/mL/h = 12.8 pmol/l/min. A positive ARR for PA using conventional units is ≥30 and using SI units is ≥60 [12,13]; ^3^ Futher details in text and Table 1.

## Data Availability

Data are unavailable due to confidentiality of patient records in compliance with HIPAA.

## References

[1] Antonelli A., Ferrari S.M., Ragusa F., Elia G., Paparo S.R., Ruffilli I., Patrizio A., Giusti C., Gonnella D., Cristaudo A. (2020). Graves’ disease: Epidemiology, genetic and environmental risk factors and viruses. Best Pract. Res. Clin. Endocrinol. Metab..

[2] Davies T.F., Andersen S., Latif R., Nagayama Y., Barbesino G., Brito M., Eckstein A.K., Stagnaro-Green A., Kahaly G.J. (2020). Graves’ disease. Nat. Rev. Dis. Primers.

[3] Smith T.J., Hegedus L. (2016). Graves’ Disease. N. Engl. J. Med..

[4] Xia Y., Kellems R.E. (2011). Receptor-activating autoantibodies and disease: Preeclampsia and beyond. Expert. Rev. Clin. Immunol..

[5] Williams T.A., Mulatero P., Bidlingmaier M., Beuschlein F., Reincke M. (2015). Genetic and potential autoimmune triggers of primary aldosteronism. Hypertension.

[6] Hannemann A., Wallaschofski H. (2012). Prevalence of primary aldosteronism in patient’s cohorts and in population-based studies--A review of the current literature. Horm. Metab. Res..

[7] Savard S., Amar L., Plouin P.F., Steichen O. (2013). Cardiovascular complications associated with primary aldosteronism: A controlled cross-sectional study. Hypertension.

[8] Young B.C., Levine R.J., Karumanchi S.A. (2010). Pathogenesis of preeclampsia. Annu. Rev. Pathol..

[9] (2020). Gestational Hypertension and Preeclampsia: ACOG Practice Bulletin, Number 222. Obstet. Gynecol..

[10] Gunatilake S.S.C., Bulugahapitiya U. (2017). Coexistence of Primary Hyperaldosteronism and Graves’ Disease, a Rare Combination of Endocrine Disorders: Is It beyond a Coincidence-A Case Report and Review of the Literature. Case Rep. Endocrinol..

[11] Okawa T., Asano K., Hashimoto T., Fujimori K., Yanagida K., Sato A. (2002). Diagnosis and management of primary aldosteronism in pregnancy: Case report and review of the literature. Am. J. Perinatol..

[12] Funder J.W., Carey R.M., Mantero F., Murad M.H., Reincke M., Shibata H., Stowasser M., Young W.F. (2016). The Management of Primary Aldosteronism: Case Detection, Diagnosis, and Treatment: An Endocrine Society Clinical Practice Guideline. J. Clin. Endocrinol. Metab..

[13] Byrd J.B., Turcu A.F., Auchus R.J. (2018). Primary Aldosteronism: Practical Approach to Diagnosis and Management. Circulation.

[14] Turchi F., Ronconi V., di Tizio V., Boscaro M., Giacchetti G. (2011). Blood pressure, thyroid-stimulating hormone, and thyroid disease prevalence in primary aldosteronism and essential hypertension. Am. J. Hypertens..

[15] Armanini D., Nacamulli D., Scaroni C., Lumachi F., Selice R., Fiore C., Favia G., Mantero F. (2003). High prevalence of thyroid ultrasonographic abnormalities in primary aldosteronism. Endocrine.

[16] Santori C., Di Veroli C., Di Lazzaro F., Caliumi C., Petramala L., Cotesta D., Iorio M., Serra V., Celi M., D’Erasmo E. (2005). High prevalence of thyroid disfunction in primary hyperaldosteronism. Recenti Prog. Med..

[17] Sabbadin C., Mian C., Nacamulli D., Dona G., Presotto F., Betterle C., Boscaro M., Bordin L., Armanini D. (2017). Association of primary aldosteronism with chronic thyroiditis. Endocrine.

[18] Tanaka M., Izeki M., Miyazaki Y., Horigome M., Yoneda T., Tsuyuki S., Takami S., Aiba M. (2002). Combined primary aldosteronism and Cushing’s syndrome due to a single adrenocortical adenoma complicated by Hashimoto’s thyroiditis. Intern Med..

[19] Krysiak R., Okopien B. (2012). Coexistence of primary aldosteronism and Hashimoto’s thyroiditis. Rheumatol. Int..

[20] Larouche V., Snell L., Morris D.V. (2015). Iatrogenic myxoedema madness following radioactive iodine ablation for Graves’ disease, with a concurrent diagnosis of primary hyperaldosteronism. Endocrinol. Diabetes Metab. Case Rep..

[21] Yokota N., Uchida T., Sasaki A., Kobayashi K., Kida O., Yamamoto Y., Eto T., Tanaka K. (1991). Thyrotoxic periodic paralysis complicated with primary aldosteronism. Jpn. J. Med..

[22] Kuo C.C., Yang W.S., Wu V.C., Tsai C.W., Wang W.J., Wu K.D. (2009). Hypokalemic paralysis: The interplay between primary aldosteronism and hyperthyroidism. Eur. J. Clin. Investig..

[23] Meyer L.S., Gong S., Reincke M., Williams T.A. (2020). Angiotensin II Type 1 Receptor Autoantibodies in Primary Aldosteronism. Horm. Metab. Res..

[24] Riemekasten G., Philippe A., Nather M., Slowinski T., Muller D.N., Heidecke H., Matucci-Cerinic M., Czirjak L., Lukitsch I., Becker M. (2011). Involvement of functional autoantibodies against vascular receptors in systemic sclerosis. Ann. Rheum. Dis..

[25] Rossitto G., Regolisti G., Rossi E., Negro A., Nicoli D., Casali B., Toniato A., Caroccia B., Seccia T.M., Walther T. (2013). Elevation of angiotensin-II type-1-receptor autoantibodies titer in primary aldosteronism as a result of aldosterone-producing adenoma. Hypertension.

[26] Li H., Yu X., Cicala M.V., Mantero F., Benbrook A., Veitla V., Cunningham M.W., Kem D.C. (2015). Prevalence of angiotensin II type 1 receptor (AT1R)-activating autoantibodies in primary aldosteronism. J. Am. Soc. Hypertens..

[27] Kem D.C., Li H., Velarde-Miranda C., Liles C., Vanderlinde-Wood M., Galloway A., Khan M., Zillner C., Benbrook A., Rao V. (2014). Autoimmune mechanisms activating the angiotensin AT1 receptor in ‘primary’ aldosteronism. J. Clin. Endocrinol. Metab..

[28] Lin J.H., Peng K.Y., Kuo Y.P., Liu H., Tan C.B., Lin Y.F., Chiu H.W., Lin Y.H., Chen Y.M., Chueh J.S. (2022). Aldosterone-producing nodules and CYP11B1 signaling correlate in primary aldosteronism. Endocr. Relat. Cancer.

[29] Turcu A.F., Auchus R. (2021). Approach to the Patient with Primary Aldosteronism: Utility and Limitations of Adrenal Vein Sampling. J. Clin. Endocrinol. Metab..

[30] Lim J.S., Rainey W.E. (2020). The Potential Role of Aldosterone-Producing Cell Clusters in Adrenal Disease. Horm. Metab. Res..

[31] Omata K., Anand S.K., Hovelson D.H., Liu C.J., Yamazaki Y., Nakamura Y., Ito S., Satoh F., Sasano H., Rainey W.E. (2017). Aldosterone-Producing Cell Clusters Frequently Harbor Somatic Mutations and Accumulate With Age in Normal Adrenals. J. Endocr. Soc..

[32] Wallukat G., Homuth V., Fischer T., Lindschau C., Horstkamp B., Jupner A., Baur E., Nissen E., Vetter K., Neichel D. (1999). Patients with preeclampsia develop agonistic autoantibodies against the angiotensin AT1 receptor. J. Clin. Investig..

[33] Walther T., Wallukat G., Jank A., Bartel S., Schultheiss H.P., Faber R., Stepan H. (2005). Angiotensin II type 1 receptor agonistic antibodies reflect fundamental alterations in the uteroplacental vasculature. Hypertension.

[34] Siddiqui A.H., Irani R.A., Blackwell S.C., Ramin S.M., Kellems R.E., Xia Y. (2010). Angiotensin receptor agonistic autoantibody is highly prevalent in preeclampsia: Correlation with disease severity. Hypertension.

[35] Hubel C.A., Wallukat G., Wolf M., Herse F., Rajakumar A., Roberts J.M., Markovic N., Thadhani R., Luft F.C., Dechend R. (2007). Agonistic angiotensin II type 1 receptor autoantibodies in postpartum women with a history of preeclampsia. Hypertension.

[36] Zhou C.C., Zhang Y., Irani R.A., Zhang H., Mi T., Popek E.J., Hicks M.J., Ramin S.M., Kellems R.E., Xia Y. (2008). Angiotensin receptor agonistic autoantibodies induce pre-eclampsia in pregnant mice. Nat. Med..

[37] LaMarca B., Parrish M., Ray L.F., Murphy S.R., Roberts L., Glover P., Wallukat G., Wenzel K., Cockrell K., Martin J.N. (2009). Hypertension in response to autoantibodies to the angiotensin II type I receptor (AT1-AA) in pregnant rats: Role of endothelin-1. Hypertension.

[38] Brewer J., Liu R., Lu Y., Scott J., Wallace K., Wallukat G., Moseley J., Herse F., Dechend R., Martin J.N. (2013). Endothelin-1, oxidative stress, and endogenous angiotensin II: Mechanisms of angiotensin II type I receptor autoantibody-enhanced renal and blood pressure response during pregnancy. Hypertension.

[39] Wenzel K., Rajakumar A., Haase H., Geusens N., Hubner N., Schulz H., Brewer J., Roberts L., Hubel C.A., Herse F. (2011). Angiotensin II type 1 receptor antibodies and increased angiotensin II sensitivity in pregnant rats. Hypertension.

[40] Barbesino G., Tomer Y. (2013). Clinical review: Clinical utility of TSH receptor antibodies. J. Clin. Endocrinol. Metab..

[41] Silva de Morais N., Angell T.E., Ahmadi S., Alexander E.K., Dos Santos Teixeira P.F., Marqusee E. (2022). Performance of Thyroid-Stimulating Immunoglobulin Bioassay and Thyrotropin-Binding Inhibitory Immunoglobulin Assay for the Diagnosis of Graves’ Disease in Patients With Active Thyrotoxicosis. Endocr. Pract..

[42] Goichot B., Leenhardt L., Massart C., Raverot V., Tramalloni J., Iraqi H., Consensus W.-G. (2018). Diagnostic procedure in suspected Graves’ disease. Ann. Endocrinol..

[43] Kawai K., Tamai H., Mori T., Morita T., Matsubayashi S., Katayama S., Kuma K., Kumagai L.F. (1993). Thyroid histology of hyperthyroid Graves’ disease with undetectable thyrotropin receptor antibodies. J. Clin. Endocrinol. Metab..

[44] Schirpenbach C., Reincke M. (2007). Primary aldosteronism: Current knowledge and controversies in Conn’s syndrome. Nat. Clin. Pract. Endocrinol. Metab..

